# Trends in Palliative Care Utilization in Deceased Veterans With Heart Failure

**DOI:** 10.1089/pmr.2023.0067

**Published:** 2023-12-21

**Authors:** Mitchell Wice, James L. Rudolph, Lan Jiang, Hal M. Edmonson, John S. Page, Wen Chih Wu, Julio Defillo-Draiby

**Affiliations:** ^1^ Center of Innovation in Long Term Services and Support, Providence VA Healthcare System, Providence, Rhode Island, USA.; ^2^ Geriatrics and Extended Care, Providence VA Healthcare System, Providence, Rhode Island, USA.; ^3^ Department of Medicine, Warren Alpert Medical School of Brown University, Providence, Rhode Island, USA.; ^4^ Center for Gerontology and Health Services Research, Brown University School of Public Health, Providence, Rhode Island, USA.; ^5^ Warren Alpert Medical School of Brown University, Providence, Rhode Island, USA.

**Keywords:** advanced care planning, death, end of life care, heart failure, palliative care

## Abstract

**Background:**

Specialist-level palliative care in the final days does not allow time to alleviate symptoms and suffering. This analysis examined the change in the time from initial specialty-level palliative care to death among Veterans with heart failure.

**Methods:**

This retrospective cohort study examined Veterans with a diagnosis of heart failure (HF) who died between 2011 and 2021. We examined the decedents from each year as a separate cohort. The primary outcome was time from specialty-level palliative care (SPC) encounter to death in the year death occurred.

**Results:**

Of the cohort (*n* = 232,079), 56.5% did not receive SPC. Specialist-level palliative care >90 days before death more than doubled from 10.1% (2011) to 26.2% (2021), and Specialist-level palliative care in the last day of life was cut from 2.5% to 0.9%.

**Conclusion:**

For Veterans with HF, specialist-level palliative care moved earlier in the disease course and has a substantial growth opportunity.

## Introduction

Approximately 6 million American adults suffer from heart failure (HF), accounting for over 800,000 hospital discharges annually. As HF prevalence nears 8 million by 2030 (3.0% of the US population), annual costs are projected to approach $70B.^1^ Atop an ∼50% 5-year mortality following diagnosis, HF's physical and psychosocial symptom burden is considerable. Patients reported a mean of nine discrete symptoms including edema, lethargy, dyspnea, syncope, depression, anxiety, spiritual distress, and a reduced health-related quality of life (HRQL).^2,3^

Specialty-level palliative care (SPC) (where a dedicated team of trained palliative care providers are consulted to assist in the care of a patient) involvement in the care of patients with HF is associated with improved HRQL, reduced symptom burden, and significant reductions in health care utilization, including a 42% reduction in the risk of rehospitalization with home-based SPC.^4^ Patients with HF can often benefit from palliative care for prolonged periods. SPC prioritizes quality of life by managing end-stage HF symptoms, advanced care planning, and a multidisciplinary care approach.^5^ Some of these tasks can be provided by primary providers, shifting as needed to more specialized care. By approaching high-disease burden this way, we can better set expectations and provide the care patients and their families want.

Although there is a lack of consensus regarding referral criteria for the involvement of palliative care in patients with heart failure, various organizations in the United States and worldwide, such as the American College of Cardiology, the International Society for Heart and Lung Transplantation, and the European Society of Cardiology, have made recommendations supporting the early integration of palliative care into HF management.^6^ Because of the lack of consensus and unfamiliarity with SPC, most SPC occurs late in the disease course. For this reason, we undertook a retrospective analysis of the timing of SPC in HF decedents within the US Department of Veterans Affairs (VA) and analyzed the results for relevant care trends over a 10-year period to help determine if a baseline penetrance level of SPC exists which can be used as a metric for systemwide improvement to increase SPC access and allow earlier SPC in the HF disease course.

## Methods

### Ethics

This study was approved by the Providence Veterans Administration Medical Center Institutional Review Board. Informed consent was waived.

### Cohort

This study examined the national population of patients with HF who received their care at the VA from January 1, 2011 to June 30, 2021. Using VA electronic medical records (EMR) we identified deceased Veterans with HF who experienced at least one inpatient admission to the VA from January 1, 2011 to June 30, 2021.

### Measurement of palliative care

We define a SPC encounter by the presence of VA stop code (351 or 353). Unique to the VHA, stop codes are mechanisms for workload credit for encounters but do not describe the makeup of an encounter. Prior studies have shown validity in using VHA stop codes to identify SPC encounters.^7^ Those with no identified palliative care stop code were considered not to have VA-delivered SPC.

### Time from palliative care to death

From the cohort, we identified veterans' dates of death from the VA Death Registry, and retrospectively examined the VA EMR for PC stop codes. Treating the earliest SPC stop code as the date of first SPC involvement, we categorized veterans into three cohorts: SPC beginning more than 90 days preceding death; SPC beginning <90 days before death; and no SPC before death. The 90-day cutoff was based on prior literature used for oncologic patients receiving SPC.^8,9^

### Variables

VA data sets were used to obtain demographic information (age, race, sex). Comorbidities were collected using International Classification of Diseases (ICD)-9 and ICD-10 codes from the year before admission. Utilization (cost, days admitted) was collected from the VA accounting system.

### Analyses

Characteristics of the cohorts were compared using standard mean difference (SMD), as prior studies have shown a preference for SMD in large populations. For reference, an SMD >0.2 is considered unbalanced. We examined all decedents within a calendar year as an individual cohort. We did not analyze or determine individual differences between the yearly cohorts as we believed the trends were self-evident.

### Outcomes

The primary outcome was time from palliative care to death in the year of death. Secondary outcomes were the number and proportion of Veterans receiving palliative care in the year of death, along with SPC encounters on the last day of life for each cohort.

## Results

Among the 232,079 Veteran decedents with HF and one or more admissions during the study period (Table 1), the mean age (standard deviation [SD]) was 76.5 (10.7) and 227,528 (98%) were men. A significant majority—172,469 (74%)—of these Veterans were White. The mean Elixhauser Comorbidity Index (SD) was 7.9 (3.4). Hypertension, diabetes mellitus, chronic lung disease, arrhythmias, electrolyte imbalances, and renal failure each had greater than 50% prevalence in this population. The mean number of SPC consultations (SD) for this population was 2.1 (6.7), and the mean total number of days hospitalized in the final year of life (SD) was 14 (20).

**Table 1.** Patient Characteristics

Variable	All (***n*** = 232,079)
Age at death, years (SD)	76.5 (10.7)
Decedents aged 18–64 years, *n* (%)	30,856 (13.3)
Decedents aged 65–74 years, *n* (%)	76,138 (32.8)
Decedents aged 75–84 years, *n* (%)	62,071 (26.7)
Decedents aged >85 years, *n* (%)	63,014 (27.2)
Female sex	2%
White	74.3%
Black	18.0%
Hispanic	7.3%
Elixhauser Comorbidity Index (SD)	7.9 (3.4)
Neurological disease	23.6%
Chronic lung disease	57.2%
Diabetes mellitus	54.5%
Chronic kidney disease	51.7%
Liver disease	15.0%
Metastatic cancer	10.0%
Obesity	20.7%
Depression	33.5%
Days hospitalized in the last year of life (SD)	14.2 (20.6)
Total cost of care in the last year of life, $ (SD)	95,123 (124,335)
Palliative Care encounters, avg. (SD)	2.1 (6.7)

SD, standard deviation.

**FIG. 1.**
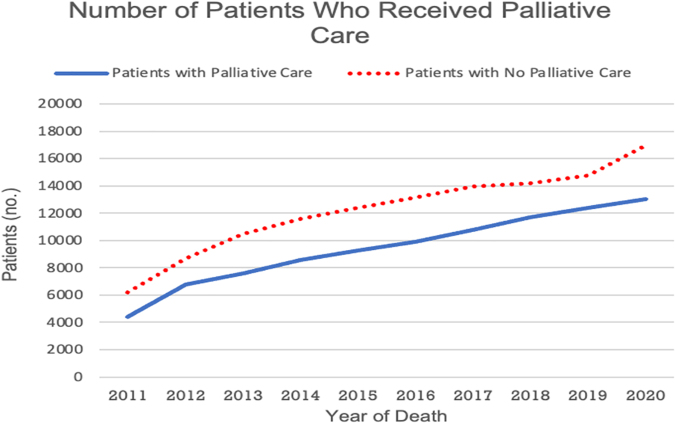
Number of patients who received or never received palliative care by year of death from January 1, 2011 to June 30, 2021.

**FIG. 2.**
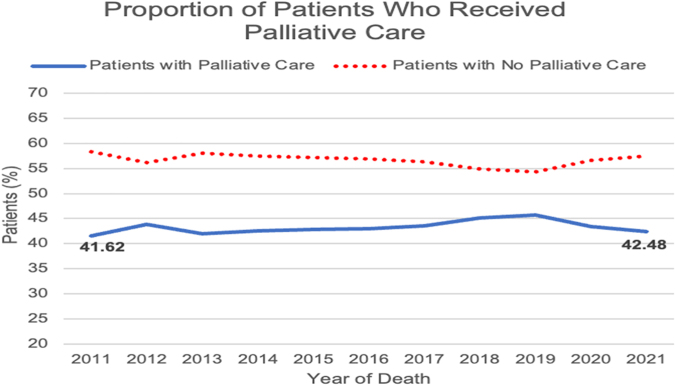
Proportion (%) of patients who either received or never received palliative care by year of death from January 1, 2011 to June 30, 2021.

**FIG. 3.**
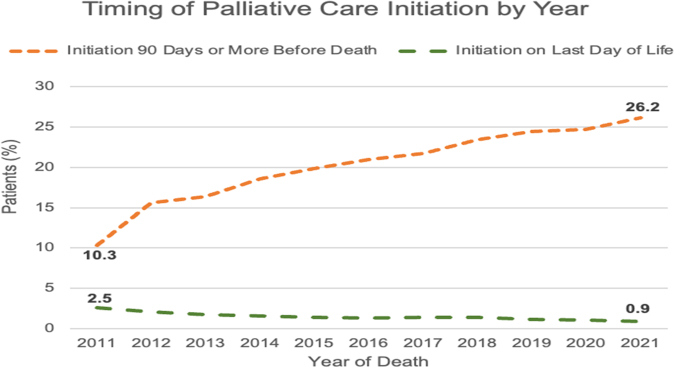
Proportion of palliative care patients who received initial palliative care either 90 or more days before death or on the last day of life between January 1, 2011 and June 30, 2021.

The time from the initial SPC encounter to death is shown in Table 2. Notably, the number of Veterans with HF and at least one admission who died each year increased annually from 10,608 in 2011 to 30,019 in 2020 (Fig. 1). Over the entire decade observed, 56.6% (*n* = 131,122) of these Veterans did not undergo a single SPC encounter at VA medical centers. The proportion of Veterans receiving any SPC increased from 41.6% (*n* = 4415) in 2011 to 42.5% (*n* = 6536) in 2021 (Fig. 2). Among Veterans that did receive SPC, the proportion with SPC involvement more than 90 days before death increased annually, from 10.31% (*n* = 1094) in 2011 to 26.2% (*n* = 4028) in 2021 (Fig. 3). The proportion with SPC initiation one or fewer days before death declined from 2.5% (*n* = 271) in 2011 to 0.9% (*n* = 140) in 2021.

**Table 2.** Timing (Days Before Death) of Palliative Care Initiation by Year (January–December)

Year	No palliative care	0–1 days	2–7 days	8–30 days	31–89 days	≥90 days	Total
2011	6193	271	921	1170	959	1094	10,608
	58.38%	2.55%	8.68%	11.03%	9.04%	10.31%	
2012	8684	314	1164	1597	1289	2413	15,461
	56.17%	2.03%	7.53%	10.33%	8.34%	15.61%	
2013	10,467	316	1239	1668	1376	2956	18,022
	58.08%	1.75%	6.87%	9.26%	7.64%	16.40%	
2014	11,556	312	1259	1706	1548	3739	20,120
	57.44%	1.55%	6.26%	8.48%	7.69%	18.58%	
2015	12,382	300	1247	1831	1604	4304	21,668
	57.14%	1.38%	5.76%	8.45%	7.40%	19.86%	
2016	13,127	304	1284	1923	1580	4832	23,050
	56.95%	1.32%	5.57%	8.34%	6.85%	20.96%	
2017	13,921	345	1241	2038	1773	5370	24,688
	56.39%	1.40%	5.03%	8.26%	7.18%	21.75%	
2018	14,207	350	1370	2038	1873	6060	25,898
	54.86%	1.35%	5.29%	7.87%	7.23%	23.40%	
2019	14,753	306	1330	2169	1963	6637	27,158
	54.32%	1.13%	4.90%	7.99%	7.23%	24.44%	
2020	16,981	314	1258	2136	1920	7410	30,019
	56.57%	1.05%	4.19%	7.12%	6.40%	24.68%	
2021^a^	8851	140	568	924	876	4028	15,387
	57.52%	0.91%	3.69%	6.01%	5.69%	26.18%	
Total	131,122	3272	12,881	19,200	16,761	48,843	232,079
	56.50%	1.41%	5.55%	8.27%	7.22%	21.05%	

^a^ Data only available for January 1 through June 30, 2021.

## Discussion

This retrospective cohort study found that over the 10-year period from January 2011 to June 2021, SPC was initiated earlier in the disease course for Veterans with HF; however, there was only minimal change in the proportion of patients receiving SPC. As HF is a disease with a high symptom burden and a nonlinear functional decline, offering SPC earlier in the disease course is essential to enhance HRQL and achieve positive outcomes for patients with HF. It is concerning that the overall percentage of veterans receiving SPC increased only modestly despite the earlier involvement of SPC. Notably, all patients were treated as inpatients in the same health care system, with eligibility and coverage determined by the same national criteria, thus removing much of the variability commonly encountered in obtaining SPC.

The trend toward earlier SPC involvement in HF cases correlates with care mandates, heightened SPC staffing, and administrative ease in SPC referrals across the VA system. As recently as 2000, only 38% of VAMCs offered inpatient SPC programs, a number which approaches 100% today following the US Department of Veterans Affairs Medical Center Hospice and Palliative Care Initiative in 2001 and the Comprehensive End-of-Life Care Initiative in 2009.^10^ These initiatives were launched in the setting of a nationwide cultural and financial recognition of SPC's benefits, as well as increasing demand by a growing population of veterans with HF being treated at VAMCs. The trend toward earlier SPC involvement with HF patients may also suggest increased recognition of SPC's benefits and willingness to consult SPC colleagues among clinicians. The minimal change in the proportion of veterans with HF who receive SPC, however, suggests that the well-documented stigma, fear, or knowledge deficits regarding SPC persist within this patient population and their families, even though the VA allows Veterans to receive disease-modifying treatments while on hospice, or concurrent care. Additionally, some patients in this study may have received treatment for and/or died of comorbid conditions, whose treating clinicians may be less familiar with SPC's availability and indications for those conditions. Finally, despite the potential for relative equalization of care within VA facilities, geographic and economic disparities remain that may make SPC less accessible to some veterans. Information on the precise staffing levels of SPC departments at individual VAMCs is also imprecise, and this data may reflect a persistent scarcity of SPC resources at some VAMCs.

The VA's unique population and care model pose limitations to the generalizability of these findings. For instance, our cohort in this study was 98% male and 74.3% White, and the VA at large serves a population that is disproportionately rural.^11^ The numerous comorbidities of many patients in this cohort and the treatment thereof may have impacted when and whether SPC was implemented. Further, an in-depth review of documentation to assess why SPC was or was not consulted for each patient and clinical course is not feasible in a sample of this size, complicating efforts to identify the discrete impacts of staffing, institutional initiatives, and changes in medical and societal attitudes upon the frequency and timing of SPC in HF patients. Furthermore, as specific outcome measures are beyond the scope of this retrospective report, it should be noted that we used SPC as a surrogate marker for quality of care as SPC has shown to be beneficial for HF patients overall.^4^ In addition, our data are from VA databases and did not look at Medicare databases which most likely results in some Veterans who received SPC or hospice in the community being miscategorized. Future studies should combine both VA and CMS databases for clearer analysis. Lastly, even though stop codes are used to signify clinical encounters and get workload credit at VA medical centers, the process can be tedious and unreliable for tracking at times. A 2019 internal VA review found that SPC was provided without the corresponding stop code in 21.5% of consults systemwide, most likely leading to an undercount of Veterans who received SPC for HF and slightly skewing data to no SPC/Hospice.^12^

## Conclusion

Our study demonstrates sustained growth in early palliative care intervention in HF patients in tandem with the rapid expansion of availability within the VA system. As an increasing number of Veterans are undergoing earlier SPC, it is hoped that they are reaping the established benefits of SPC for a longer period of time thus improving the quality of care of these Veterans, which should strengthen the case for continued expansion of SPC staffing and involvement in the clinical care of patients with HF. Further inquiry is needed to determine the barriers to increasing the persistently static proportion of Veterans with HF who receive palliative care and how they might be surmounted. Such knowledge could ultimately benefit the quality of life of all patients who suffer from this disease and lead to high-quality collaboration between cardiology and palliative care.

## Abbreviations Used

EMR: electronic medical records
HF: heart failure
HRQL: health-related quality of life
SMD: standard mean difference
SD: standard deviation
SPC: specialty-level palliative care
VA: US Department of Veterans Affairs
